# A Proposed Selection Index for Jersey Cattle in Zimbabwe

**DOI:** 10.1155/2013/148030

**Published:** 2013-03-27

**Authors:** Edward Missanjo, Venancio Imbayarwo-Chikosi, Tinyiko Halimani

**Affiliations:** ^1^Malawi College of Forestry and Wildlife, Private Bag 6, Dedza, Malawi; ^2^Department of Animal Science, Faculty of Agriculture, University of Zimbabwe, P.O. Box MP 167, Mount Pleasant, Harare, Zimbabwe

## Abstract

A multitrait selection index (*I*_*T*_) for Zimbabwean Jersey cattle was constructed. The breeding objective was defined in terms of production and functionality traits. The production component of the index included milk yield (*M*), butterfat yield (*F*), protein yield (*P*), butterfat percent (*F*%), and protein percent (*P*%), while the functional component included the somatic cell count (SCC). The index was termed as *I*_*T*_ = 0.0004*M* + 0.0109*F* + 0.0313*P* + 1.0004*F*% + 2.4491*P*% − 0.1905SCC. The accuracy of the index was 91.1%, and the correlation between this index and the aggregate breeding objective was 0.954. A selection index is more important in the selection of sires and cows. This leads to the greatest genetic progress and hence productivity in the dairy sector. Therefore, the application of the selection index developed is necessary if the dairy cattle industry is to maximise the exploitation of genetics and to improve its relative competitive position.

## 1. Introduction

König and Swalve [1] presented a multiple correlation method of constructing optimum selection indexes. However, to solve the simultaneous equations, the genetic parameters (heritability and genetic correlations) and phenotypic parameters (standard deviation and correlations) among traits must be known. When these traits differ in variability, heritability, and in the correlation among their phenotypes and genotypes, index selection is more effective than independent culling levels or sequential selection [2], and the construction of an index is not easy without the use of matrix methods, particularly, if there are more than two sources of information, and improves as the number of traits in the selection index increases [3].

Dekkers [4] reported that the selection of both production traits (protein yield, protein %) and functional traits (longevity, milkability, and somatic cell score) increased the selection index efficiency to 58%. Sørensen et al. [5] found that the selection of milk yield, somatic cell score, udder depth, teat placement, and foot angle improved efficiency of response in the aggregate genotype by 1% to 4% over selection for milk yield alone. Sun et al. [6] reported that for improving milk yield, selection indices comprising milk, fat, or protein yields were 98%–100% as efficient as an index comprising all three traits. Selection on milk yield alone was 5% less efficient in improving milk yield compared with the selection using an index of all three traits.

According to [7], every country should develop its selection index because the success of selection index from different countries cannot be compared, even though breeding goals are very similar. For some time in Zimbabwe, the only means of selection of local bulls have been on the basis of pedigree information and visual appraisal, which with no doubt had an adverse on genetic progress. Essentially, two of the most heavily used Zimbabwean bulls at one time had an average predicted difference of −370 kg milk [8]. Therefore, the objectives of this study were to develop a multitrait selection index for Jersey cattle in Zimbabwe and to test the accuracy and efficiency of the index. The index would give farmers an option of selecting one or more traits at a time, depending on the farmer's selection goals.

## 2. Materials and Methods 

### 2.1. Environment

Zimbabwe is located in southern Africa in the tropical savannah region. The total land area is 390,759 km^2^ and it is divided into five agroecological regions. Rainfall patterns and crop production progressively deteriorate from Region I to V. However, livestock production including dairying is practised in all the regions. In the regions with low rainfall, dairying is assisted by the production of drought resistant fodder crops. Most dairy farms are located within 40 km of the major cities and towns [9]. 

### 2.2. Data and Data Edits

The standard 305-day milk production records of pure bred Jersey were obtained from Zimbabwe Livestock Identification Trust (LIT). Missanjo [10] described the dataset and the edits. This gave a dataset of 10,986 records with cows calving in the period 1996–2008.

### 2.3. Statistical Analysis

Multivariate analyses using repeatability animal model and the ASReml program developed by [11] were used. The animal model included fixed effects of herd-year-season, previous calving interval, days dry, linear, and quadratic regression coefficients of age at calving as covariates, permanent environmental, and animal effects. From this, variance components, heritability, predicted breeding values (EBVs), and genetic and phenotypic correlations were estimated. These were then used in the construction of the index. SelAction programme Version 2.1 developed by [12] was used to develop the index.

The index was developed in order to allow breeders to select sires and dams for simultaneous improvement of both production and functionality traits. The production component of the index included milk yield (*M*), fat yield (*F*), protein yield (*P*), fat percent (*F*%), and protein percent (*P*%), while the functional component included the somatic cell count (SCC).

The selection index constructed was

(1) $$\begin{array}{c} Pb=G_{12}a. \end{array}$$

The selection index weights were then calculated as

(2) $$\begin{array}{c} b=P^{-1}G_{12}a, \end{array}$$

where *G*_12_ is an *n* × *m* genetic variance-covariance matrix for *m* traits affecting profitability and *n* correlated indicator traits (criteria) and incorporates the additive genetic relationships between sources of information; **P** is a *n* × *n* phenotypic (co)variance matrix of correlated indicator traits; **b** is a vector of index weights (coefficients) for the phenotypic values of the selection criteria (traits); **a** is an *n* × 1 vector which express the relative importance by the breeder to each trait. In this case, as described by [13], an equal change in standard-deviation units was used for each trait. Therefore, the “**a**” that were assigned to each trait were the reciprocals of the phenotypic standard deviations.

As stated above, the optimum set of the selection index coefficients are those which maximise the correlation (*r*_*HI*_) or minimise the squared deviation between the selection index and the aggregate genotype (breeding objective). Therefore, according to [14], the accuracy of index selection is a function of the correlation (*r*_*HI*_) between the aggregate genotype and the index and was calculated as

(3) $$\begin{array}{c} r_{HI}^{2}=\frac{\sigma_{I}^{2}}{\sigma_{H}^{2}}, \end{array}$$

where **σ**_*I*_^2^ and **σ**_*H*_^2^ were the variances of the index and the breeding objective, respectively. These variances were calculated as

(4) $$\begin{array}{c} \sigma_{I}^{2}=b'G_{12}\;a,\\ \sigma_{H}^{2}=a'G_{22}\;a, \end{array}$$

where **a** and *G*_12_ are as described above; *G*_22_ is the *m* × *m* genetic variance-covariance matrix of the *m* traits in the breeding objective. 

## 3. Results and Discussion

The selection index developed was

(5) IT=0.0004M+0.0109F+0.0313P+1.0004F%+2.4491P%−0.1905SCC.

These means that animals can then be ranked according to these index values and selection based on these rankings. The positive signs for production traits and negative sign for functionality trait mean that the index developed will allow breeders to select sires and dams, which will be increasing the production traits and decreasing the functionality trait, respectively. 

The use of SCC for selection purposes has been widely discussed because the elevation of SCC with an infection is a clear indication of the occurrence of infection as well as of the immunological response to combat the infection. Recent studies clearly indicate, however, that the genetic correlation with clinical cases of mastitis is reasonably high and that the relationship is linear. Low SCC follows low prevalence of mastitis, and high SCC indicates high prevalence. Thus, the validity of using SCC as an indirect measure to improve mastitis resistance has been strengthened [5]. 

The variances (**σ**_*I*_^2^) of this index (*I*_*T*_) and (**σ**_*H*_^2^) of the breeding objectives were computed as 12.23 and 13.43, respectively. The accuracy of the index was 91.1%, and the correlation with the objective was 0.954.

To test the effect of individual criteria on the efficiency of the index (*I*_*T*_), these criteria were deleted one at a time from the index. The efficiency of these sub-indices was then compared to the efficiency of the overall index. These results are summarized in Table 1.

Table 1 clearly shows that most individual criteria have only a small influence on the efficiency of the index. However, when milk yield, fat yield, or protein yield is dropped from the index, the resultant subindices are only 51.7%, 53.3%, or 52.9% accurate, respectively, compared to the 91.1% of the total index with these criteria included. Since dropping certain traits has a small influence on the efficiency of the index, the possibility to construct the total index without these traits (criteria) was investigated. However, when criteria were dropped from the index, the index weights (coefficients) of the remaining criteria changed. For instance when *P*% or *F*% was dropped from the index the index weights (*b*-values) for milk yield and SCC changed from positive to high negative value and from negative to high positive, value respectively, implying decreased milk yield and increased SCC, which is unacceptable. The same thing happened when *F*% and *P*% were both dropped at the same time from the index. It was, therefore, decided to retain the total index.

## 4. Conclusion

In this study, a detailed description of the development of a multitrait selection index was presented. Although this index was developed specifically for the Jersey cattle breed in Zimbabwe, the method employed can be used to develop indices for different breeds and different production systems within the same breed. The definition of the breeding objective and correlation structure between traits and criteria is necessary. Application of these principles and results is necessary if the dairy cattle industry is to maximise the exploitation of genetics and to improve its relative competitive position. 

## Figures and Tables

**Table 1:** Reduction in the accuracy of the subindex, compared to the total index (*I*_*T*_), when individual or some criteria(s) were dropped from the index.

	*I*_*T*_	Criteria							
		*M *	*F*	*P *	*F*%	*P*%	SCC	*F*% and *P*%	*F*%, *P*%, and SCC
*r*_*HI*_^2^	0.911	0.517	0.533	0.529	0.893	0.907	0.868	0.835	0.871
Reduction	—	0.394	0.378	0.382	0.018	0.004	0.043	0.076	0.040

*I*_*T*_: total Index, *M*: milk yield, *F*: fat yield, *P*: protein yield, *F*%: fat percent, *P*%: protein percent, SCC: somatic cell count, and r_*HI*_^2^: index accuracy.
